# Ag nanoparticles immobilized sulfonated polyethersulfone/polyethersulfone electrospun nanofiber membrane for the removal of heavy metals

**DOI:** 10.1038/s41598-022-09802-9

**Published:** 2022-04-06

**Authors:** Md Eman Talukder, Md. Nahid Pervez, Wang Jianming, George K. Stylios, Mohammad Mahbubul Hassan, Hongchen Song, Vincenzo Naddeo, Alberto Figoli

**Affiliations:** 1 Shenzhen Institute of Advanced Technology, Chinese Academy of Sciences, Shenzhen, 518055 China; 2 University of Chinese Academy of Sciences, Beijing, 100049 China; 3 Guangdong Key Lab of Membrane Material and Membrane Separation, Guangzhou Institute of Advanced Technology, Guangzhou, 511458 China; 4 Sanitary Environmental Engineering Division (SEED), Department of Civil Engineering, University of Salerno, via Giovanni Paolo II 132, 84084 Fisciano, SA Italy; 5 Research Institute for Flexible Materials, School of Textiles and Design, Heriot-Watt University, Galashiels, TD1 3HF UK; 6 Fashion, Textiles and Technology Institute, University of the Arts London, 20 John Prince’s Street, London, W1G 0BJ UK; 7 Institute On Membrane Technology (CNR-ITM), University of Calabria, 87036 Rende, CS Italy

**Keywords:** Environmental sciences, Materials science

## Abstract

In this work, *Eucommia ulmoides* leaf extract (EUOLstabilized silver nanoparticles (EUOL@AgNPs) incorporated sulfonated polyether sulfone (SPES)/polyethersulfone (PES) electrospun nanofiber membranes (SP ENMs) were prepared by electrospinning, and they were studied for the removal of lead (Pb(II)) and cadmium (Cd(II)) ions from aqueous solutions. The SP ENMs with various EUOL@AgNPs loadings were characterized by X-ray diffraction (XRD), Fourier transform infrared (FTIR) spectroscope, thermo-gravimetric analysis (TGA), scanning electron microscopy (SEM), transmission electron microscopy (TEM), and contact angle (CA) measurements. The adsorption studies showed that the adsorption of Cd(II) and Pb(II) was rapid, achieved equilibrium within 40 min and 60 min, respectively and fitted with non-linear pseudo-second-order (PSO) kinetics model. For Cd(II) and Pb(II), the Freundlich model described the adsorption isotherm better than the Langmuir isotherm model. The maximum adsorption capacity for Cd(II) and Pb(II) was 625 and 370.37 mg g^−1^ respectively at neutral pH. Coexisting anions of fluoride, chloride, and nitrate had a negligible influence on Cd(II) removal than the Pb(II). On the other hand, the presence of silicate and phosphate considerably affected Cd(II) and Pb(II) adsorption. The recyclability, regeneration, and reusability of the fabricated EUOL@AgNPs-SP ENMs were studied and they retained their high adsorption capacity up to five cycles. The DFT measurements revealed that SP-5 ENMs exhibited the highest adsorption selectivity for Cd(II) and the measured binding energies for Cd(II), Pb(II), are 219.35 and 206.26 kcal mol^−1^, respectively. The developed ENM adsorbent may find application for the removal of heavy metals from water.

## Introduction

According to recent updates of the World Health Organization (WHO) and United Nations International Children’s Emergency Fund (UNICEF), almost more than 2.2 billion people worldwide cannot manage pure drinking water^1,2^. The scarcity of pure drinking water is a global threat that occurs due to rapid deforestation and intense industrialization. Pollution of surface and groundwater by heavy metals is a serious issue, which can affect human health as it can cause many serious diseases including cancer, and pollutes our environment. Many heavy metals exist in surface and groundwater (such as Pb, Cd, Cr, Cu, Ni, As, and Zn) due to discharging untreated water from various industrial processes and human activities^3–6^. Among them, Pb(II) and Cd(II)\are the most common water pollutants with extreme toxicity as they are harmful to body tissue even at low concentrations^7,8^, which is of serious concern for public safety and health^9,10^. Because of their severe toxicity to aquatic organisms and humans, heavy metal pollution has caused significant concern. Hence, before discharging toxic heavy metal enriched effluents into the environment, they need to be processed and decontaminated by a viable and efficient method. Up until presently, several techniques have been used to remove heavy metals from wastewater, such as chemical precipitation, adsorption, reverse osmosis, electrodeposition, nanofiltration, microfiltration, and ultrafiltration^11,12^. Among all methods, adsorption is widely used for removing heavy metal ions because of its low operational cost, high effectiveness, simplicity, and low cost.

In the case of heavy metals removal by adsorption, the number of adsorption sites, functional groups, surface area, chemical nature, and polarity are the key essential properties that can influence the binding of pollutants by an adsorbent^13^. ENMs are very effective in removing heavy metals from contaminated water and show high heavy metal removal efficiency than any other conventional absorbents due to their exceptionally high surface area^14–17^. Electrospinning is the easiest and highly versatile technique, which allows the fabrication of one-dimensional fibrous ENMs adsorbent with different properties, such as very high surface area, high porosity, and tailored mechanical properties^18–20^. According to previous studies, the PES-ENMs are highly effective for removing heavy metals from contaminated water. Some functionalized composite polymer ENMs or nanoparticle-immobilized ENMs were used, such as poly(acrylic acid) (PAA)/poly(vinyl alcohol) (PVA)^21^, polyvinylpyrrolidone (PVP)/SiO_2_^22^, PAA thermally cross-linked with PVA fibrous ENMs, to remove Cu^2+^ ions from contaminated water^23^. Other ENMS studied for the removal of heavy metals from water may include PES−TiO_2_^24^, poly(vinyl chloride)^25^, and aminated polyacrylonitrile^26^.

The PES-ENMs have been studied for the removal of heavy metals due to their superior thermal and mechanical properties, and also due to their resistance against various chemicals^24^. However, PES-ENMs are still not a good adsorbent for the removal of heavy metal ions from aqueous solutions as their surface is highly inert. Therefore, several methods have been employed to improve the adsorption capacity of PES-ENMs, such as surface modifications and graft-copolymerization^24,27^. On the other hand, the SPES polymer shows high adsorption capacity due to the presence of the sulfonate moieties that bind positively charged metal ions^28^. Previous studies show that the addition of small amounts of metallic nanoparticles to electrospun polymeric nanofibers improves their metallic ion absorption capacity^29–31^. Sulfonate groups are known to act as active adsorption sites for various metals ions, such as Pb(II), Cu(II), Zn(II)^32,33^, Cd(II), and Cu(II)^34^ helping their removal from aqueous solutions. It was reported that the incorporation of AgNPs to polymeric ENMs showed high removal of Cu(II), Ni(II), Pb(II), Hg(II), and Zn(II) ions^35–37^.

The quantum computation using density functional theory (DFT) and the prediction of the experimental adsorption selectivity in terms of metal-oxygen binding energy measurements of metal ion adsorption by various adsorbents can be made. In our adsorption study, DFT has been used to explore the geochemical processes and interactions between adsorbates and adsorbents^38^, particularly among different kinds of metal oxides or hydrated metal oxides. By using DFT quantum computation, Bashir et al. have determined the experimental adsorption selectivity in terms of metal-oxygen binding energy measurements of Zn(II), Pb(II), Cd(II), and Hg(II) ions and confirmed that Zn(II), Pb(II), Cd(II), and Hg(II) ions bind into the inner-sphere of the adsorbent with the edge/corner-sharing tridentate (ECT) complex which is the most favorable adsorption configuration^39–41^. Chen et al. studied the adsorption of Cu(II), Pb(II), Co(II), and Ni(II) by MnFe_2_O_4_ surfaces involving chemical bonds and H-bond, at neutral pH using DFT calculations^42^. To the best of our knowledge, this study for the first time explains the novel adsorption mechanism of Pb(II), Cd(II) on different aspects of typical EUOL@AgNPs incorporated SP-ENMs via DFT calculations.

The present study investigates the fabrication of EUOL@AgNPs incorporated SP-ENMs prepared by the electrospinning method for the removal of Pb(II) and Cd(II) ions from aqueous solutions. The role of the sulfonate groups and EUOL@AgNPs to improve adsorption performance was demonstrated in this research work. The effect of changing different operating parameters such as pH, contact time, initial concentration of metal ions, and temperature on the metal ion adsorption, and regeneration and recycling to obtain the maximum adsorption conditions were investigated.

## Materials and methods

In this research, polyethersulfone (PES), Ultrason E6020P (average M_w_: 6 58 kDa,), is purchased from BASF (Germany). Dimethylacetamide (DMAc) was purchased from TNJ Chemical Industry Co., Ltd, China. Polyvinylpyrrolidone, (PVP)-K30, was purchased from Chemical Reagents Co., Ltd, China. Sulfonated polyethersulfone, SPES, (5% sulfonation), was purchased from Changzhou Kete Chemical co., Ltd, China. Silver nitrate solution and glucose were purchased from Aladdin-Reagent Co., Ltd (China). Sodium hydroxide (NaOH) and acetic acid (CH_3_COOH) were obtained from Sinopharm Chemical Reagent Co., Ltd. (China). Dry *Eucommia ulmoides* leaf was purchased from Xian faithful Biotechnology Co., Ltd (China). Non-woven PET paper was supplied by Guocheng CO. (China), and Standard Pb(II) and Cd(II) ion solutions were purchased from the national standard sample library (1000 µg mL^−1^ containing 1.0 mol L^−1^ HNO_3_ (1.0 mol L^−1^ nitric acid). All polymers and auxiliaries were used without further purification.

### Preparation of *Eucommia ulmoides* leaf extract powder

*Eucommia ulmoides* leaf powder (100 g) was extracted using a Soxhlet extractor with 500 mL of petroleum ether under reflux conditions for 2 h. The separation of the extract was carried out by filtration with Whatman No. 2 filter paper. The extraction was evaporated to 50 mL volume by a vacuum at below 40 °C using a rotary evaporator (Model R52; Shanghai Yarong Biochemistry Instrument Co., Shanghai, China), dried in an oven at 45 °C or dried naturally, and stored at − 4 °C until further use.

### Fabrication of *Eucommia ulmoides* leaf extract (EUOL)-mediated Ag nanoparticles

As shown in Fig. S1, silver nitrate solution was prepared by adding 3.4 g of AgNO_3_ to 20 mL distilled water in a conical flux. The PVP solution was prepared by dissolving PVP, glucose, and NaOH in 60 mL distilled water. The mixed solution was stirred for 10 min and then EUOL powder was added to the PVP solution, and heated at 60 °C under continuous stirring. AgNO_3_ solution was then added to EUOL containing PVP solution drop-by-drop. The mixed solution was stirred for 10 min at 50 °C. The nanoparticles were separated by centrifugation, and the solid products were washed with distilled water several times until no NO_3_^-^ could be traced. And then, EUOL@AgNPs were stored in the refrigerator for further use.

### Fabrication of SP and EUOL@AgNPs loaded SP ENMs

The SP-1 and SP-2, 3, 4, 5 ENMs were produced using the electrospinning technique (Table 1). A solution of SP was prepared by dissolving 8% PES (w/v) and 6% SPES (w/v) to 86 mL DMAc under stirring for 4 h at room temperature. ENMs were produced using an electrospinning machine (Model: M06, Foshan Lepton Precision Measurement And Control Technology Co., Ltd, China) on a PET nonwoven sheet placed on a rotating drum collector for at least 2 h. Each solution was injected at a feeding rate of 0.8 mL h^−1^ under an applied voltage of 16.5 kV at 28 °C. The distance between the needle tip and the rotating drum collector was adjusted to 18 cm. The obtained ENMs thickness was between 0.4 and 0.5 mm. The formed ENMs were separated from the PET nonwoven sheet and the retained solvent in the ENMs is removed by drying in a vacuum oven for 5 h at 60 °C. EUOL@AgNPs (wt%: 0.5, 1.5, 2.5, and 4.0%) is loaded on the SP-1 ENMs by dispersion. The solution was magnetically stirred for approximately 3 h to obtain a homogenous solution. The prepared solutions SP-2, 3, 4, 5 ENMs were collected in 20 mL syringes, and each solution and ENMs were produced using the same electrospinning machine under the same conditions mentioned above.

**Table 1:** EUOL@AgNPs percentage for EUOL@AgNPs-SP-ENMs preparation.

Sample code	Figure code	Nanoparticles amount	ENMs samples
SP-1	a_1_, b_1_	EUOL@Ag-SP (0.0 wt%)	SPES/PES ENMs
SP-2	a_2_, b_2_	EUOL@Ag-SP (0.5 wt%)	EUOL@Ag-SP-ENMs-1
SP-3	a_3_, b_3_	EUOL@Ag-SP (1.5 wt%)	EUOL@Ag-SP-ENMs-2
SP-4	a_4_, b_4_	EUOL@Ag-SP (2.5 wt%)	EUOL@Ag-SP-ENMs-3
SP-5	a_5_, b_5_	EUOL@Ag-SP (4.0 wt%)	EUOL@Ag-SP-ENMs-4

### Characterization

The SP solution viscosity was measured using a Rotational Viscometer (NDJ-8S Digital Viscosity Meter, Movel Scientific Instrument Co., Ltd, China) at room temperature. The electrical conductivity and the pH of the SP solution were measured by a DZS-706A Multi-parameter Analyzer (Shanghai INESA Scientific Instrument Co., Ltd., China) at ambient temperature. The contact angle of the ENMs sample was assessed by using a contact angle measurement apparatus (Model: Dropmeter A-300, Kudos Precision Instruments, and the USA). The morphology of the ENMs was examined by scanning electron microscopy (SEM) (Phenom XL, Phenom world, Thermo Scientific, Japan) at an accelerating voltage of 5 kV. Energy-dispersive X-ray spectroscopy (EDX) was used to confirm the quality of polymer dispersion in the solvent and to identify the presence of EUOL@AgNPs on the ENMs surface using an SEM equipped with an EDX attachment (Model: 4500H EDXRF spectrometer, Skyray Instrument, Massachusetts, USA). The size of Ag nanoparticles was measured by Transmission Electron Microscopy (TEM) and the TEM images were acquired using a Tecnai G2 20 TEM (Thermo Fisher Scientific, USA) under an N_2_ atmosphere**.** The ENMs were further characterized by X-ray Powder Diffractometry and XRD patterns were recorded on an Empyrean X-ray Diffractometer, Malvern PANalytical, United Kingdom) from 10° to 80° 2θ range at a rate of 5° min^−1^. The ENM’s functional groups were identified using a Fourier transform infrared spectrometer (Model: Interspec 200-X, Interspectrum, Estonia) from 400 to 4000 cm^−1^ and 64 scans were signal-averaged. The ENMs thermal decomposition behavior was studied using a thermogravimetric analyzer (TG 209 F1 *Libra* Netzsch, Germany) under an N_2_ atmosphere.

### Heavy metals adsorption by ENMs

The calibration curve was prepared by measuring various concentrations of standard Pb(II) and Cd(II) solutions and the prepared calibration curve is provided in Fig. S3 (Supplementary Materials). The limit of detection (LOD) and limit of quantification (LOQ) for heavy metal measurement by ICP-OES were 6 and 12 µg L^−1^ respectively^43^.

### Effect of solution pH

The effect of pH on the heavy metal adsorption by the ENMs was carried out by adding 25 mg ENMs adsorbent to 20 mL of 250 µg mL^−1^ heavy metal solution. The pH of the solution was adjusted using dilute acetic acid (CH_3_COOH) for an acidic pH and sodium hydroxide (NaOH) solution for an alkaline pH. The effect of adsorbent dosage, pH, and initial concentration were investigated. The final equilibrium Pb(II) and Cd(II) ion concentrations were analyzed by the ICP-OES instrument, MILESTONE, ETHOS 1, Model: optima 8000 American Perkin Elmer Company, USA. 

### Adsorption experiments

The adsorption performance of the SP-1 and SP-2, 3, 4, 5 ENMs were investigated. In this study, a constant amount of ENMs adsorbent (25 mg) was added to 10 mL aqueous solution containing various concentrations of Pb(II) and Cd(II). Pb(II) and Cd(II) solutions containing ENMs adsorbents were placed in a rotary shaker (Electric Decolorizing Orbital Shaker Ts-2000A, Shanghai, China) at 25 °C room temperature. This study investigated the effect of the initial concentrations of Pb(II) and Cd(II) ions on the equilibrium adsorption by SP-1 as well as SP-2, 3, 4, 5 ENMs. Five different initial concentrations of Pb(II) and Cd(II); 250, 300, 350, 400, 450 µg mL^−1^ were used. The equilibrium curves were studied at room temperature (25 °C). For studying the equilibrium curves, 25 mg of SP-1 and SP-2, 3, 4,5 ENMs adsorbents were added into a 10 mL aqueous solution containing 250, 300, 350, 400, 450 µg mL^−1^ of Pb(II) and Cd(II), as previously reported by Côrtes et al.^44^. These samples were placed in a rotary shaker at room temperature and agitated at 220 rpm. Kinetic curves with contact times ranging from 0 to 100 min, an initial Pb(II) and Cd(II) concentration of 250 µg mL^–1^, a temperature of 25 °C, a stirring velocity of 220 rpm, and appropriate adsorbent dose and pH values were investigated. The same kinetics experiment has been done by Côrtes et al.^45^ and Dotto et al.^44,46^.

The concentrations of Pb(II) and Cd(II) were monitored at different time intervals (15 min, 30 min, 50 min) using an ICP-OES instrument (Model: Optima 8000, Perkin Elmer Corporation, USA) at different temperatures. The following relationships were used to determine the percentage removal of Pb(II) and Cd(II) ions (% removal) (Eq. 1) and equilibrium adsorption capacity (mg g^−1^) (Eq. 2)^47,48^.

1 $$ \% Heavy\,metal\,removal = \frac{C_{subs0} - C_e}{C_{subs0}} \times 100 $$

2 $$ Adsorption\, capacity, qe = \frac{C_{subs0} - C_{e}}{M} \times V{ } $$

where C_subs0_ and C_e_ are the initial and equilibrium concentrations of metal ions in solution (mg L^−1^), respectively, V is the total solution volume (mL), and M is the mass of the ENMs adsorbent (mg).

### Competing for coexisting ion experiment

The five anions (phosphate (PO_4_^3−^), chloride (Cl^−^), nitrate (NO_3_^2−^), silicate (SiO_3_^2−^), and fluoride (F^−^)) with 0.1–0.5 mM concentration were used to investigate the effect of coexisting anions on Pb(II) and Cd(II) adsorption by the ENMs. The 250 µg mL^−1^ concentration of Pb(II) and Cd(II) and the 25 ppm concentration of competing for coexisting ion solutions were used.

### ENMs adsorbent regeneration studies

The adsorbent regeneration studies were employed for Pb(II) and Cd(II) removal by adding 25 mg of adsorbent to 10 mL Pb(II) and Cd(II) solutions (250 µg mL^−1^) in a glass bottle. After 2 h of shaking at 220 rpm, each adsorbent was withdrawn, washed thoroughly with distilled water, added to a new 10 mL 0.05 M concentration NaOH solution, and shaken for 2 h. Finally, the adsorbent was again washed with deionized distilled water, dried, and then added to a new 10 mL Cd(II) and Pb(II) solutions. The recycling and regeneration of ENMs were carried out ten times.

###  Quantum chemical calculations

Theoretical calculations were performed on a model material, using density functional theory (DFT) as incorporated in the Gaussian 03 set of codes. Geometry optimizations were carried out with Becke’s three-parameter hybrid model using the Lee–Yang–Parr correlation function (B3LYP) and LanL2DZ basis set.

## Results and discussion

In this study, nanoparticles were immobilized into SP ENMs due to their high surface area and good thermal properties. In recent years, hydrophilic ENMs have been widely studied for wastewater treatment. However, super hydrophilic ENMs might become the next potential super adsorbent, as supported by the results of our study which fabricated nanoparticles that immobilized super hydrophilic SP- ENMs with high levels of Pb(II) and Cd(II) adsorption. At the end of our experiments, the adsorption kinetics and isotherm equations were studied for the removal of heavy metals.

### Physical properties of SP solutions

The physical properties of a polymer solution are the most crucial factor to form nanofibers in the electrospinning process. The viscosity and electrical conductivity of a polymer solution are the most decisive parameters that influence the morphology of the produced ENMs. The solution parameters of SP are listed in Table 2. After nanoparticle immobilization, the viscosity of the SP solution increased linearly from 2465 to 2658 mPa S^−1^, and its electrical conductivity increased from 1.4 to 1.5 µS cm^−1^, respectively. It has been reported that the shape and size of EUOL@AgNPs affect the electrical conductivity^49,50^. In general, the increased electrical conductivity of the polymer solution is increased with an increase in the concentration of EUOL@AgNPs^50^. The diameter of nanofiber decreases with an increase in the electrical conductivity of the polymer solution^51^.

**Table 2:** Physical properties of the SP solution.

Sample	Viscosity (mPa S^-1^)	Electric conductivity (µS cm^-1^)	Diameter (nm)
SP-1 ENMs	2465	1.40	69.9 ± 16.01
SP-2 ENMs	2498	1.45	74.3 ± 18.34
SP-3 ENMs	2527	1.45	79.5 ± 19.27
SP-4 ENMs	2601	1.48	78.1 ± 19.01
SP-5 ENMs	2658	1.50	83.8 ± 21.54

### Morphologies of ENMs

**Figure 1:**
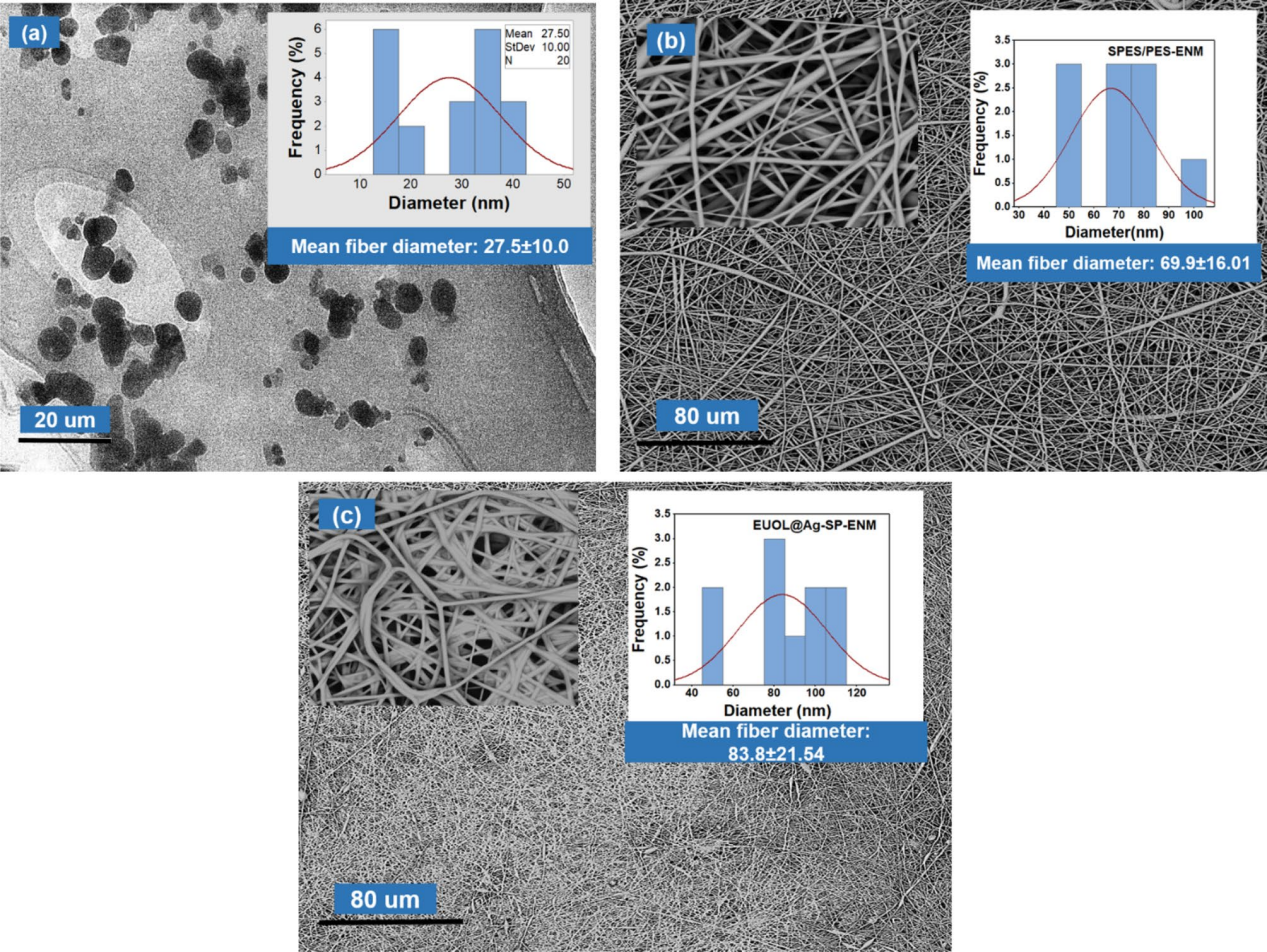
(**a**) TEM image of EUOL@AgNPs and SEM micrographs of (**b**) SP-1, and (**c**) SP -5 ENMs.

The size and morphology of synthesized EUOL@AgNPs were investigated using TEM. As shown in TEM micrographs (Fig. 1a), homogeneously distributed EUOL@AgNPs with a round shape are formed and the average particle size of synthesized EUOL@AgNPs was found to be 102.8 ± 56.9 nm. The surface morphologies of the SP-1 and SP-5 ENMs were analyzed using SEM. Figure 1b, c represent the SEM images of SP-1 and SP-5 ENMs. The SP-1 ENMs have shown a uniform structure with an average fiber diameter of approximately 69.9 ± 16.01 nm. After the incorporation of EUOL@AgNPs, the fiber diameter of SP-5 slightly increased to approximately 83.8 ± 21.54 nm along with increased surface roughness, as shown in Fig. 1c. The surface roughness change is occurred due to the presence of EUOL@AgNPs, which increased the solution viscosity and electrical conductivity, which is consistent with previous studies^47,52^.

### Hydrophilicity

The hydrophilicity of the produced ENMs was studied by contact angle measurement and water adsorption. As shown in Fig. S2a, both types of ENMs showed high water uptake. From the result of water uptake behavior, it is evident that EUOL@AgNPs immobilized SP ENMs showed better hydrophilicity compared to the SP-1 ENMs. The static water contact angle of SP ENMs was 15.6° but the static water contact angle exhibited by the EUOL@AgNPs immobilized SP-5 ENMs was 9.7°, as shown in Fig. S2b, c. Polyphenols are highly hydrophilic due to the presence of abundant hydroxyl groups and therefore the EUOL@AgNPs immobilized SP ENMs showed slightly higher hydrophilicity compared to the SP-1 ENMs.

### EDX characterization

**Figure 2:**
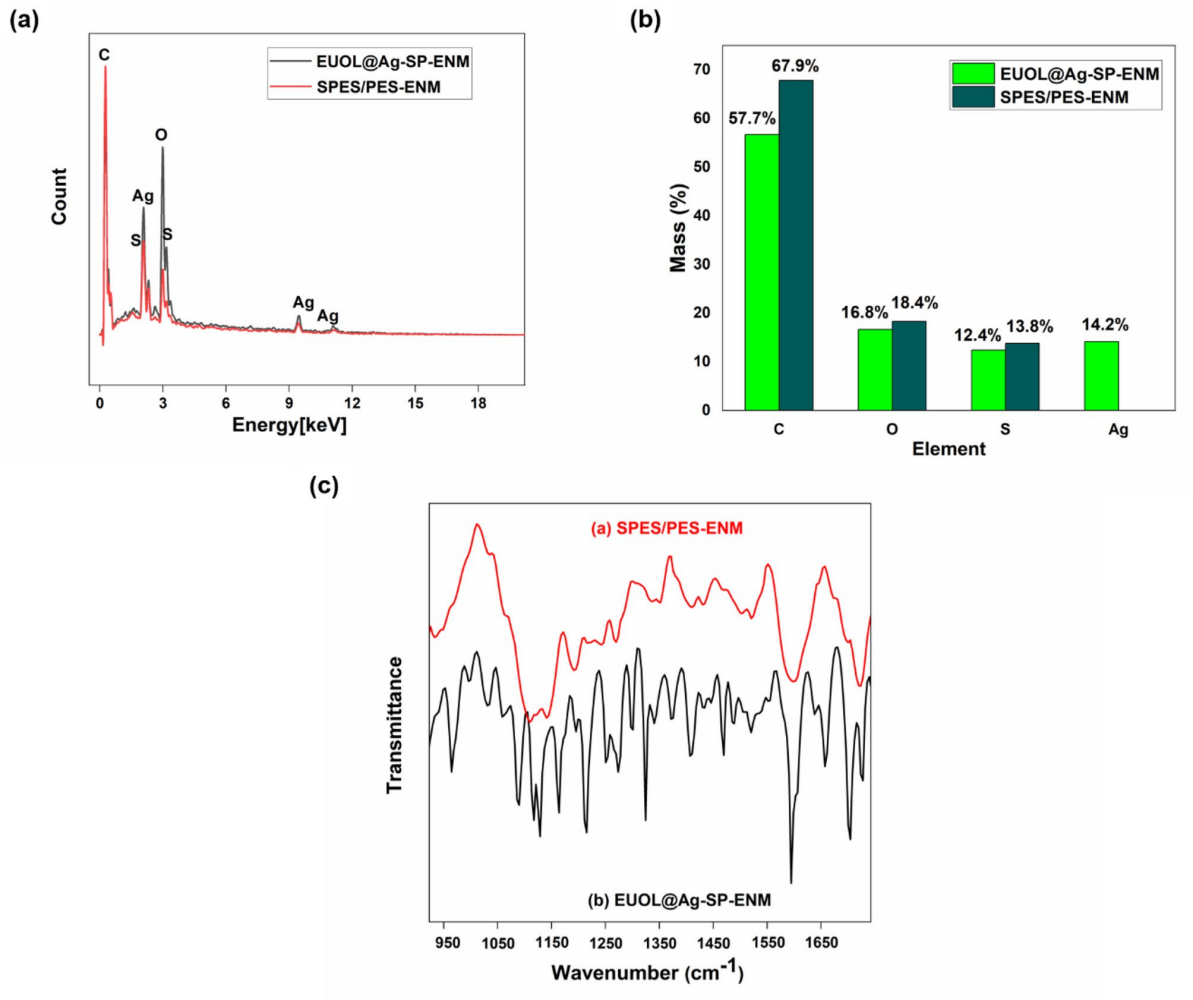
(**a**, **b**) EDX of SP-1 and SP-5 ENMs, and (**c**) FTIR spectra of SP-1 and SP-5 ENMs.

The elemental composition of SP-1 and SP-5 ENMs was investigated by EDX analysis. It was observed that the control SP-1 ENMs only contained a high amount of carbon, oxygen, sulfur, while the EUOL@Ag-NPs immobilized SP ENMs showed an additional signal of silver (Fig. 2a**).** The EDX spectrum of control SP-1 ENMs does not show any signal for the Ag. The highest silver content (14.2%) was shown by the SP-5 ENMs (Fig. 2b). The EDX analysis confirms the presence of EUOL@AgNPs in the SP ENMs (SP-5).

Figure 2c shows the FTIR spectra of the control SP-1 and SP-5 ENMs. The adsorption peak at 1145 cm^−1^ is assigned to symmetric O=S=O stretching vibration due to the -SO_2_ groups in the PES polymer macromolecular chains. The adsorption bands at 1038 cm^−1^ and 1300 cm^−1^ are related to the stretching vibration of –SO_3_H groups, which indicates the presence of SPES in the ENMs^53^. Figure 2c shows the FTIR spectra of the SP-5 ENMs. The absorption peaks at 925 cm^−1^ and 1676 cm^−1^ are attributed to the stretching vibration of EUOL@Ag-OH. The absorption band at 1042 cm^−1^ is related to the EUOL@Ag–O–Ag@EUOL asymmetrical stretching vibration^54^. Thus, the higher the amount of EUOL@AgNPs added, the greater the intensity of the absorption band observed. These absorption bands indicate that EUOL@AgNPs were successfully incorporated into the polymer matrices. 

### X-ray diffraction (XRD) analysis

**Figure 3:**
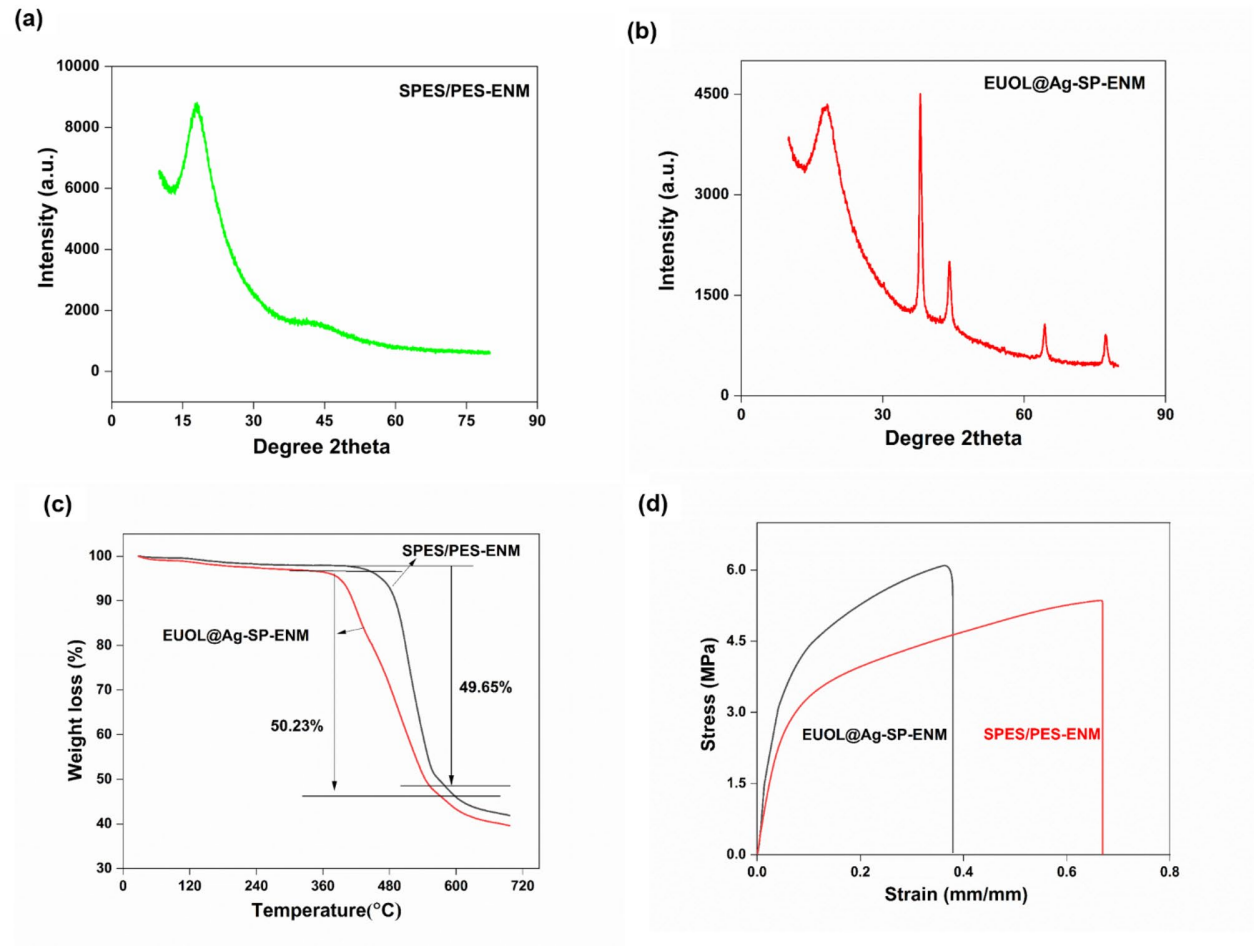
XRD results of (**a**) SP-1 ENMs, and (**b**) SP-5 ENMs (**c**) TGA results of SP-1 and SP-5 ENMs, and (**d**) Tensile strength of SP-1 and SP-5 ENMs.

The chemical compositions and crystallinity of SP-1 and SP-5 ENMs were analyzed by XRD. As shown in Fig. 3a, the XRD pattern of SP-1 ENMs shows an amorphous wide broad peak at 2θ values of 16°–19° because of the presence of the rigid benzene ring and the flexible ether bond, suggesting that the formed ENMs is amorphous. On the other hand, the SP-5 ENMs not only exhibited a broad amorphous peak at 2θ values of 16°–19° but also sharp peaks at 2θ values of 37.92°, 44.21°, 64.43°, and 77.3°, which can be Miller Indexed as (1 1 1), (2 0 0), (2 2 0), and (3 1 1) diffractions as shown in Fig. 3b, that are related to the EUOL@AgNPs. Similar peaks were observed by others for the EUOL@AgNPs-loaded SP ENMs^55,56^. The presence of the amorphous peaks suggests that the addition of EUOL@AgNPs to SP did not change the ENMs' amorphous structure.

### TGA analysis

The thermal stability of the SP-1 and SP-5 ENMs were carried out by thermo-gravimetric analysis (TGA), as shown in Fig. 3c. The SP-1 ENMs exhibited four-stage thermal degradation at room temperature; 120, 120–450, 450–560, and 560–800 °C. The first stage of weight loss was due to the loss of absorbed moisture, but the maximum weight loss occurred in the third stage due to the degradation of SPES and PES macromolecular chains. In the final stage, more degradation occurred of the degraded components produced. The SP-5 ENMs also exhibited four-stage thermal degradation at room temperature; 120, 120–360, 360–550, and 550–800 °C but the maximum degradation temperature decreased, suggesting that the addition of EUOL@AgNPs to SP solution decreased its thermal stability. The major decomposition of SP-ENM started from 415 °C but after the incorporation of EUOL@AgNPs to its, its decomposition started from 362 °C, and residual ash content decreased from 50.35 to 47%. This could be due to EUOL@AgNPs catalysed the break down of SP-ENM structure at high heat^57,58^.

### Tensile strength analysis

The mechanical properties of ENMs have been reported to be crucial for practical applications in adsorption studies. The Young’s modulus and tensile strength of SP-1 and SP-5 ENMs were calculated from stress and strain curves as shown in Fig. 3d. The SP-1 ENMs showed good mechanical strength with a tensile strength of 5.4 MPa and elongation of 69.66%. The incorporation of EUOL@AgNPs to SP slightly increased the tensile strength and also elongation of the produced ENMs, showing EUOL@AgNP's interaction with the macromolecular chains of SP^39^. The tensile strengths, Young modulus, and elongation values of both ENM are shown in Table 3. The SP-1 ENMs showed higher elongation (69.6%) than EUOL@AgNPs-SP-ENMs (37.9%). SP-5 ENMs have a higher tensile strength of 6.1 ± 1.0 MPa than the SP-1 ENMs.

**Table 3:** Young modulus, tensile strength, and elongation of samples.

Membrane type	Young’s modulus (MPa)	Tensile strength (MPa)	Elongation (%)
SP-1 ENMs	8.02	5.4	69.6
SP-2 ENMs	15.2	5.8	35.4
SP-3 ENMs	15.5	5.84	36.7
SP-4 ENMs	16.3	5.9	36.9
SP-5 ENMs	16.3	6.1	37.9

### Heavy metal adsorption study

The adsorption kinetics of Pd(II) and Cd(II) adsorption by SP-1, and SP-2, 3, 4, 5 ENMs were investigated using a series of experiments. The effect of solution pH and initial concentrations of heavy metals on the adsorption process was studied. The adsorption isotherm models were also examined.

#### Calibration curve of heavy metal

When an electron multiplier, particularly on the dynode, is influenced, ICP-OES shows ion and acts as a detector. When Cd(II), Pb(II) ions interact with the dynode, electrons are released and a detectable pulse is produced^59,60^. The ICP-built-in OES's software then compares the intensities and creates a calibration curve. The calibration curve was created in this work for ICP-OES analysis to determine the precise concentration of heavy metal ions in the solution^45,60^. As illustrated in Fig. S3, the linear line represents the value of R^2^ at 0.99.

#### Effect of pH

**Figure 4:**
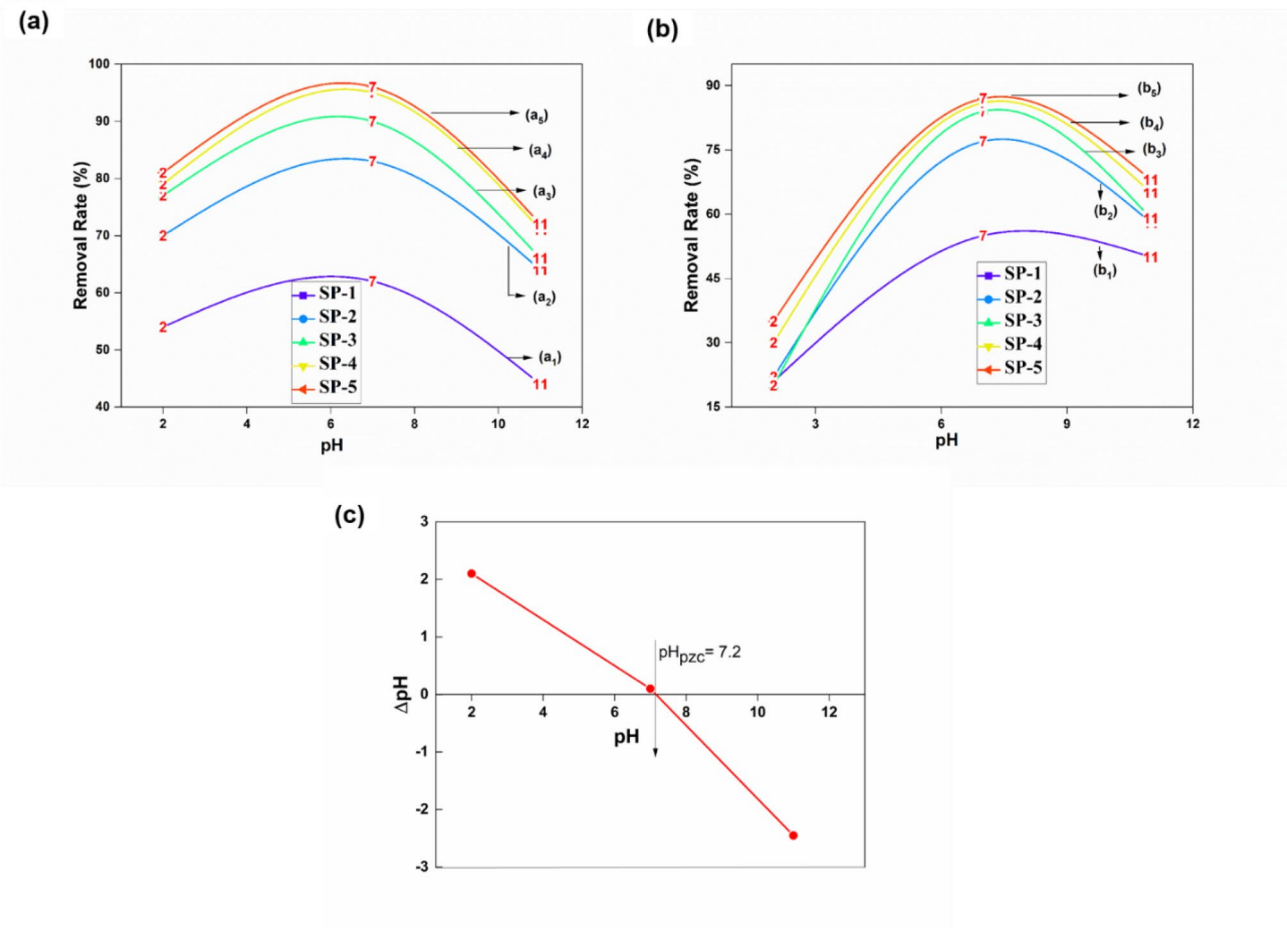
Effect of pH on adsorption removal of (**a**) Cd(II), and (**b**) Pb(II) by SP-1 and SP-2, 3, 4, 5 ENMs, (**c**) pH_pzc_ plot of SP-5 ENMs.

The solution pH is one of the most crucial factors that directly changes the adsorbate surface and influences the adsorption process. Therefore, the effect of solution pH on Cd(II), Pb(II) adsorption by the SP-1 and SP-2, 3, 4, 5 ENMs were evaluated at a wide range of pH (2–11), and results are presented in Fig. 4a, b. At a pH range of 6.5–7.5, the Cd(II) removal by SP-5 ENMs reached 94–97%, as shown in Fig. 4a and for Pb(II), the removal efficiency was 85–88% by SP-5 ENMs from an aqueous solution., as shown in Fig. 4b. The effect of pH on the Pb(II) adsorption was minor for the EUOL@AgNPs-SP-ENMs, while it was significant for Cd(II) adsorption in pH-7.0–7.5. The EUOL@AgNPs-SP-ENMs sulfonate group acts as a ligand by keeping a delicate balance between hydrogen and metal ion coordination in uranyl ion complexes. By blending EUOL@AgNPs with SP ENMs the adsorption capacity percentage is increased. The same phenomena happened in previous few studies^35,61–63^. The adsorption capacity percentage rises gradually by Increasing EUOL@AgNPs. Hydrogen bonding involving OH (water), S=O groups as donors is a dominant component that acts as a medium to strong interactions; whilst the sulfonate groups are frequent acceptors. Hydrogen bonding of coordinated sulfonates is recognized in various metal ion complexes, as revealed in previous studies^64,65^.

The effect of pH on heavy metal absorption by SP-5 ENMs could be explained in terms of pHpzc of the adsorbent in the solution (pHpzc = 7.2, Fig. 4c), as Cd(II), Pb(II) adsorption would be facilitated by electrostatic interaction between positively charged Cd(II), Pb(II) species (H_2_CdO_3_^−^ and H_2_PbO_3_^−^ are predominant in the experimental pH range) and negatively charged absorbent surface, are shown in Fig. 4c. In this case, the higher removal efficiency was due to the abundant protonation of the adsorption sites on SP-5 ENMs, which interacted with positively charged metal ions species at pH < pHpzc. With the increasing pH, the net surface charge on the adsorbent became less positive and even negative, and repulsive forces between cations adsorbate and adsorbent resulted in a decrease of the Cd(II), Pb(II) adsorption capacity^43,45^.

#### Effect of initial concentration

**Figure 5:**
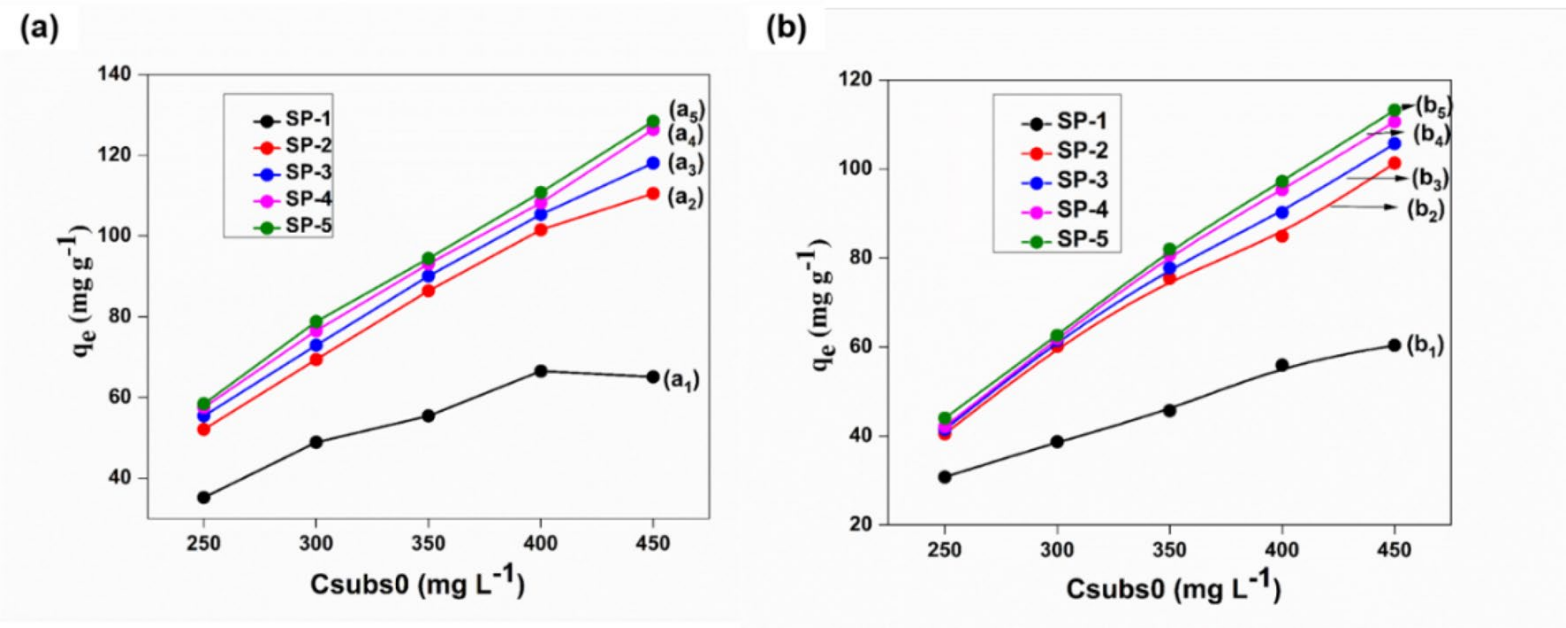
The adsorption capacity of initial concentration of (**a**) Cd(II), and (**b**) Pb(II) by SP-1 and SP-2, 3, 4, 5 ENMs.

The effect of the initial concentration of Cd(II) and Pb(II) ions on the binding capacities of the SP-1 and SP-2, 3, 4, 5 ENMs are shown in Fig. 5. For the EUOL@AgNPs-SP-ENMs, adsorption capacity rapidly increased with an increase in initial concentration of Cd(II) and Pb(II)*,* whereas, for SP-1 ENMs, the ENMs was saturated with Cd(II) and Pb(II) at Csubs0 of 400 mg L^−1^ as the adsorption reached a plateau but for SP-5 ENMs, the Cd(II) and Pb(II) binding capacity was still increasing. As shown in Fig. 5a, the Cd binding of 128.4 mg g^−1^ was achieved for the SP-5 ENMs compared to the SP-1 ENMs, which showed the Cd(II) binding capacity of 65.1 mg g^−1^ at the initial concentration of 450 mg L^−1^. Similarly, the Pb(II) adsorption of 113.2 mg g^−1^ was achieved for the SP-5 ENMs compared to the 60.4 mg g^−1^ achieved for the SP-1 ENMs, which showed the Pb(II) binding capacity at the initial concentration of 450 mg L^−1^, as shown in Fig. 5b. The addition of cationic EUOL@AgNPs to SP considerably enhanced the heavy metal binding capacity. It’s shown that increased adsorption capacity with the increasing of EUOL@AgNPs percentage.

#### Adsorption kinetics

**Figure 6:**
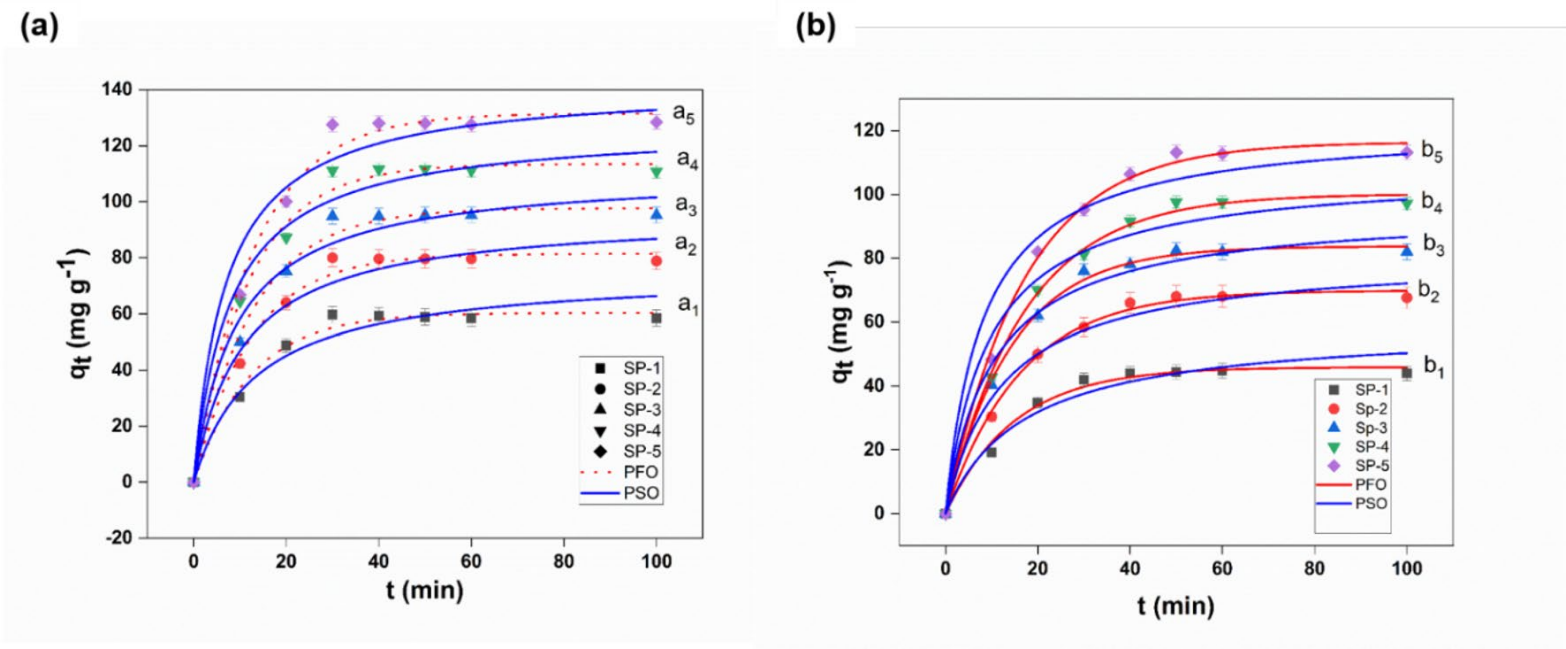
Kinetic curves onto SP-1, and SP-2, 3, 4, 5 ENMs; [Metal_(Csubs0)_ = 0.2 mg L^−1^, at pH-7, 220 rpm for 100 min, at room temperature for (**a**) Cd(II), and (**b**) Pb(II). (Adjustment of non-linear PFO and PSO kinetic models to the experimental data).

The Pb(II) and Cd(II) ions are toxic and typically found in contaminated surfaces and groundwater. These are very harmful to human health. Many scientists have paid a great deal of attention to the removal of Pb(II) and Cd(II) because of the severe Pb(II) and Cd(II) pollution in recent years. However, the adsorbent material needs to exhibit rapid and stable adsorption kinetics in removing heavy metal ions from the aqueous solution^45,66^. Therefore, in this experiment, the adsorption kinetic models were investigated by placing 25 mg of SP-1 and SP-2, 3, 4, 5 ENMs in a 10 mL solution of 250 µg mL^−1^ Pb(II), and Cd(II) concentration^66^. The effect of contact time (5–100 min) on the adsorption of Pb(II) and Cd(II) ions was investigated, as shown in Fig. 6.

Adsorption of Cd(II) occurred rapidly and reached 80% at approximately 20 min; 100% equilibrium adsorption was obtained in 40 min. By contrast, 80% of the adsorption of Pb(II) occurred comparatively slowly, and reached 100% equilibrium adsorption within 60 min. The mechanism is already explained previously in the pH 7 section.

For SP-2, 3, 4, 5 ENMs the adsorption capacity progressed rapidly and almost reached equilibrium within 30 min. On the other hand, for the pure SP-1 ENMs, the adsorption capacity was lower, and equilibrium reached within 45 min, as shown in Fig. 6. The adsorption capacities increased gradually regardless of the Pb(II), and Cd(II) concentration. More than 90% of the total adsorption of Pb(II), and Cd(II) occurred within the first 60 min for SP-2, 3, 4, 5 ENMs, which was much higher than pure SP-1 ENMs.

The adsorption behavior of SP-1 and SP-2, 3, 4, 5 ENMs for Pb(II) and Cd(II) ions is investigated, and adsorption kinetic data fitness is examined with two commonly used adsorption kinetic models. The Non-linear PFO model and PSO model were applied to describe the kinetic data comparison between SP-1 and SP-2, 3, 4, 5 ENMs, as shown in Fig. 6. Figure 6a, b illustrates that the non-linear PFO and PSO curve produces the best fitting results for both Pb(II) and Cd(II) ions. The PFO was found to be the most appropriate for describing the kinetic behaviour than the PSO model. But the value for the coefficient of determination (R^2^) and adjusted coefficient of determination (R^2^_adj_) (between 0.98 and 0.98) and the lowest values for the average relative error (ARE < 1.4%) are lower values for PFO than the PSO. The PFO model indicates that the adsorption mechanism is dependent on the diffusion-controlled system. For Cd(II), the maximum adsorption capacities for SP-5 and SP-1 ENMs were 142.09 mg g^−1^ and 75.14 mg g^−1^, respectively. For Pb(II), the maximum adsorption capacities for SP-5 and SP-1 ENMs were 121.9 mg g^−1^, and 59.6 mg g^−1^, respectively.

The mechanism of Pb(II) and Cd(II) elimination was investigated using the PFO, PSO kinetic models. Sorption is preceded by diffusion across a boundary layer in the PFO model. Chemical surface adsorption, where the removal of adsorbate from a solution is due to chemical interactions between the adsorbent and the adsorbate, is the rate-limiting step in the PFO model. Also, the high concentration used can indicate that kinetics followed the non-linear PFO model. To analyze the adsorption kinetics precisely, PFO (Eq. 3) and PSO (Eq. 4) models were used, which can be expressed as follows.

3 $$ Q_{t} = Q_{e} (1 - e^{{ - k_{1} t}} ) $$

4 $$ Q_{t} = \frac{{k_{1} Q_{e} 2t}}{{1 + k_{2} Q_{e} t}} $$

where Q_t_ (mg g^−1^) and Q_e_ (mg g^−1^) are the adsorption capacity at any time and adsorption equilibrium, the parameters k_1_ (min^−1^) and k_2_ (g mg^−1^ min^−1^) are the first and second-order kinetic rate constants, respectively.

Many researchers have been reporting similar results for the adsorption kinetics of various pollutants onto PES–TiO_2_^−^ ENMs, magnetite/graphene oxide hybrid (MGO)^67^. Compared to other similar ENMs adsorbents studied, the SP-5 ENMs studied in this work showed much higher Cd(II) and Pb(II) adsorption. The improved adsorption performance of SP-5 ENMs could be related to the increase in adsorption sites on the ENMs surface due to the addition of EUOL@AgNPs.

The experimental qe values obtained from the PFO kinetic model are shown in Table 4. The qe values for Pb(II) and Cd(II) ions increased with an increase in the EUOL@AgNPs loading. Here, the SP-1 and SP-2, 3, 4, 5 ENMs have both shown better removal of Cd(II) than Pb(II).

**Table 4:** Adsorption kinetic parameters for Cd(II), and Pb(II) uptake onto SP-1, and SP-2, 3, 4, 5 ENMs for non-linear PFO and PSO.

	Adsorption of Cd (‖I)						Adsorption of Pb (‖I)				
Adsorbent ENMs		K1, K2 (1/min)	Qe (mg g-1)	R^2^	R2adj	ARE %	K1, K2 (1/min)	Qe (mg g-1)	R2	R2adj	ARE %
SP-1	PFO	0.08	60.32	0.99	0.99	1.4	0.067	45.95	0.98	0.98	1.3
	PSO	0.001	75.14	0.96	0.96	1.9	0.001	59.6	0.96	0.95	1.4
SP-2	PFO	0.08	81.44	0.99	0.99	1.6	0.06	69.85	0.99	0.99	1.03
	PSO	0.001	95.79	0.97	0.97	2.0	0.001	80.98	0.98	0.98	1.4
SP-3	PFO	0.07	97.69	0.99	0.99	1.9	0.069	83.70	0.99	0.99	0.99
	PSO	0.001	110.76	0.97	0.97	2.2	0.001	96.74	0.98	0.98	1.5
SP-4	PFO	0.08	113.45	0.99	0.99	2.1	0.058	100.13	0.99	0.99	1.2
	PSO	0.001	127.07	0.98	0.98	2.4	0.001	107.69	0.97	0.97	2.4
SP-5	PFO	0.07	131.56	0.99	0.99	2.7	0.58	116.40	0.99	0.99	1.1
	PSO	0.001	142.09	0.96	0.96	3.4	0.001	121.90	0.96	0.96	3.3

#### Adsorption isotherms

**Figure 7:**
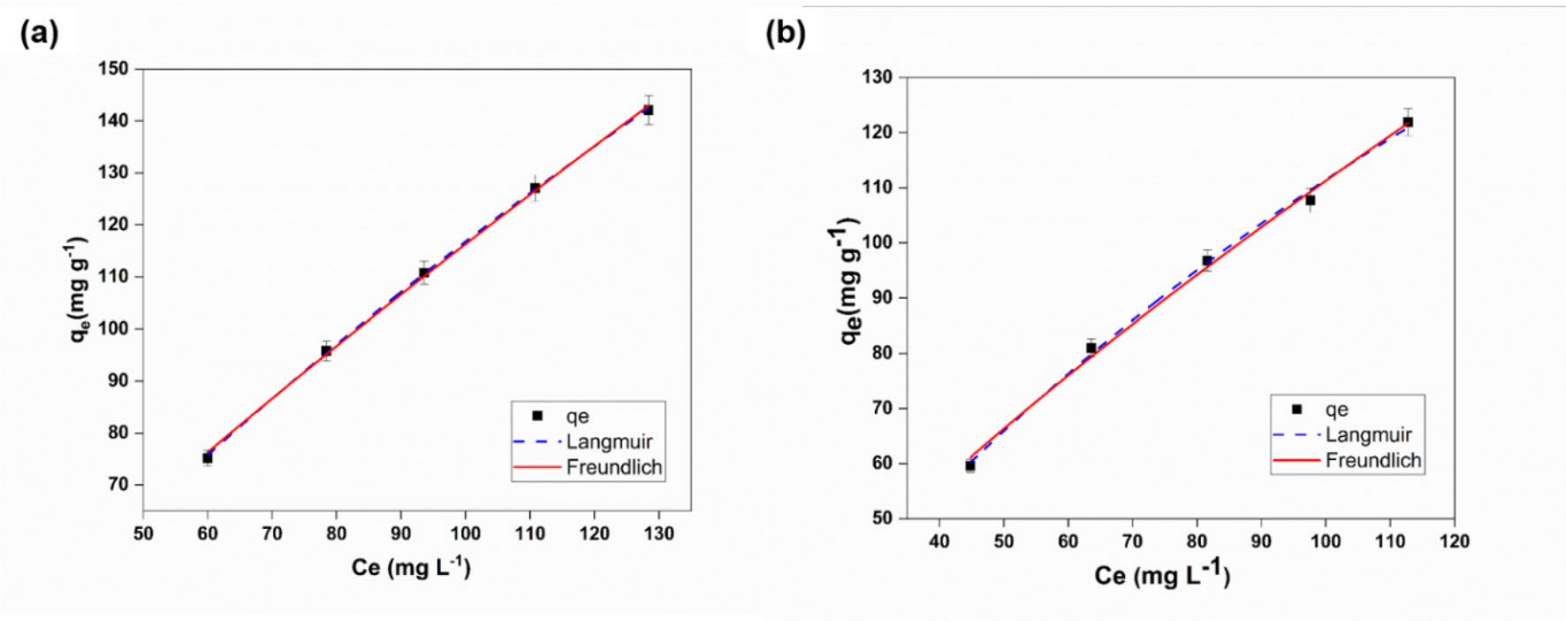
Equilibrium curves of Langmuir and Freundlich isotherm model for (**a**) Cd(II), and (**b**) Pb(II) adsorption onto SP-5 ENMs. [Metal_(Csubs0_ = 0.2 mg L^−1^, pH = 7, 2 h, and at room temperature.

Figure 7a, b shows the Cd(II), and Pb(II) adsorption capacity of SP-1 and SP-2, 3, 4, 5 ENMs. Two of the adsorption isotherms models were used to describe the interactive behavior between ENMs adsorbents and heavy metals. The interaction behavior between adsorbent and adsorbate can be simulated by applying the well-established fundamental Langmuir and Freundlich isotherm models. The Langmuir isotherm model represents monolayer adsorption, and the Freundlich isotherm model represents multilayer adsorption^11,43^. The Pb(II) and Cd(II) ions adsorption isotherms for SP-5 ENMs, are shown in Fig. 7. The SP-5 ENMs showed a maximum adsorption capacity of 625 mg g^−1^ for Cd(II) and 370.37 mg g^−1^ for Pb(II) at neutral pH. It demonstrates that the SP-5 ENMs have shown considerably higher Pb(II) and Cd(II) ions adsorption capacity at neutral pH.

Adsorption isotherms Pb(II) and Cd(II) are well known, Langmuir (Eq. 5) and Freundlich (Eq. 6) isotherm models are used to eanalyze the adsorption equilibriums data, which can be expressed as follows.

5 $$ Q_{e} = \frac{{Q_{m} k_{L} C_{e} }}{{1 + k_{L} C_{e} }} $$

6 $$ Q_{e} = \, k_{F} C_{e}^{1/n} $$

where C_e_ (mg L^−1^) and Q_e_ (mg g^−1^) are the amounts of the equilibrium concentration and equilibrium adsorption quantities, and k_F_ (mg g^−1^)*(L mg^−1^)^1/n^ is Freundlich constant, and 1/n is the heterogeneity factor. 

As shown in Table 5, after analyzing the adsorption data, the correlation coefficients (R^2^), R^2^adj, and ARE (%) values indicate that the Langmuir model has a good fitting and shows a higher regression co-efficient value than the Freundlich model. Cd(II) and Pb(II) adsorption on SP-5 ENMs has better fitting by the Langmuir isotherm model, indicating a monolayer adsorption mechanism. From this point of view, our prepared novel adsorbent α- exhibited a greater adsorption capacity for heavy metals than reported before (Table 6),

**Table 5:** Parameters of Langmuir and Freundlich models for Cd(II) and Pb(II) adsorption isotherm on SP-5 ENMs.

Metal	Adsorbent	Langmuir					Freundlich isothermal				
		Q_max_ (mg g^-1^)	k_L_ (L mg^-1^)	R^2^	R^2^_adj_	ARE (%)	k_F_	1/n	R^2^	R^2^_adj_	ARE (%)
Cd(II)	SP-5 ENMs	625	0.0023	0.999	0.99	0.21	2.6	0.82	0.998	0.998	1.07
Pb(II)	SP-5 ENMs	370.37	0.0043	0.997	0.996	2.07	3.5	0.75	0.996	0.995	2.8

**Table 6:** Comparison of SP-5 ENMs with other ENMs.

ENMs type	Adsorption capacities (mg g-1)		References
	Cd(II)	Pb(II)	
PAN/Fe_3_O_4_@Fe_3_O_4_	–	57.0 ± 4	^68^
PEO/Chitosan nanofiber membrane	232.3	214.8	^13^
sodium alginate and Poly(Vinyl alcohol) nanofiber	163.9	–	^69^
PVA/PAA nanofiber	159.0	102.0	^70^
PVA/CS	148.8	266.1	^69^
Cellulose Acetate Modified with poly(glycidylmethacrylate and grafted with polyacrylic acid	160.0	–	^71^
PES–TiO_2_ Electrospun Mats	–	∼ 1.3	^24^
PAN Crosslink, amination phosphorylation	7.4	73.3	^72^
SP-5 ENMs	625	370.37	This work

### Effect of competing anions

**Figure 8:**
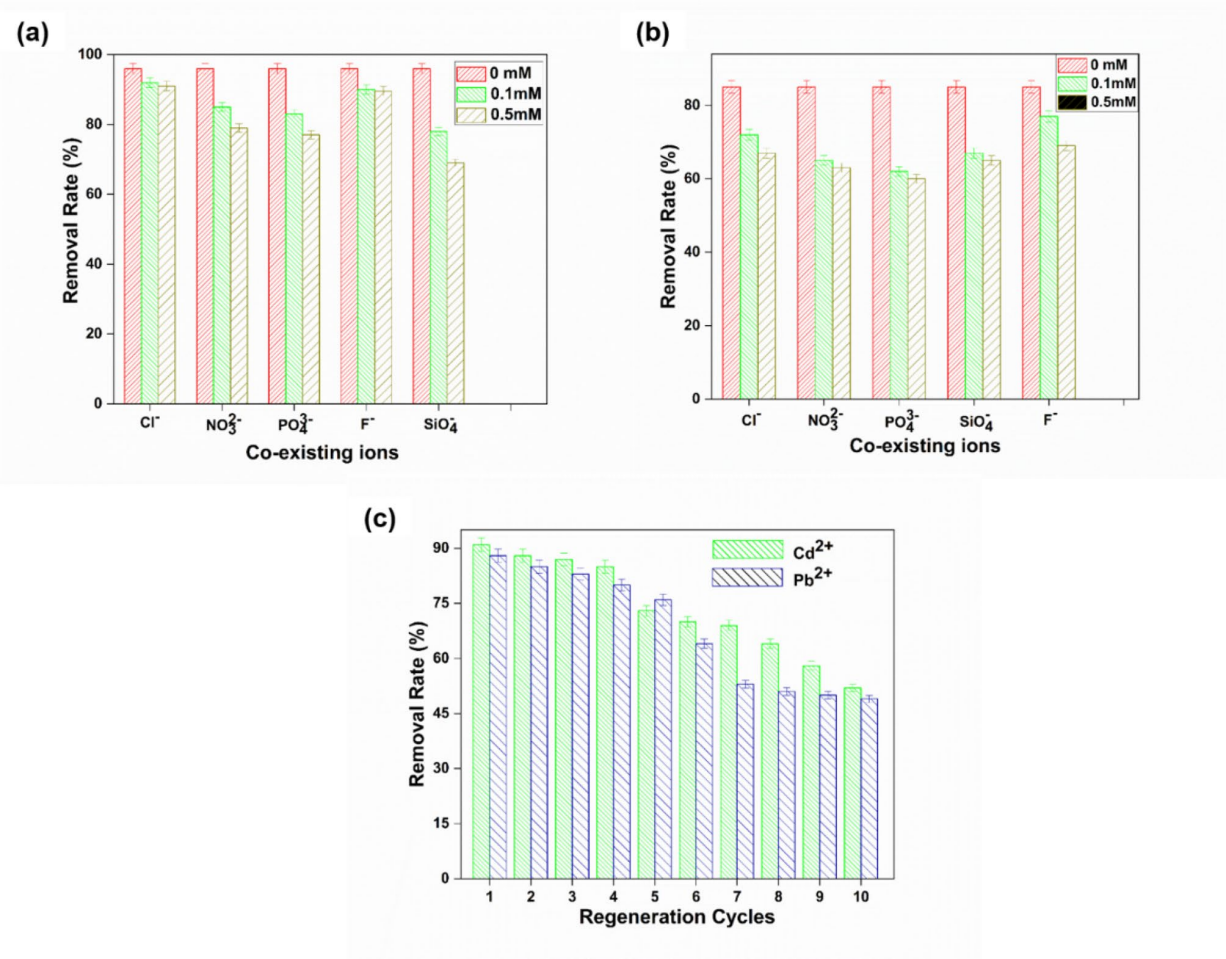
Effect of coexisting ions on the (**a**) Cd(II), and (**b**) Pb(II) adsorption by SP-5 ENMs, and (**c**) recycle and reusability performance of the SP-5 ENMs for Cd(II), and Pb(II) removal.

Here, the effect of coexisting anions were examined for only SP-5 ENMs adsorbent. Various anions are present in polluted water and many natural water sources, and these anions can affect the Cd(II) and Pb(II) removal by blocking some of the adsorption sites of the adsorbent, thus, competing with Cd(II) and Pb(II) ions. Here, the effect of F^−^, Cl^−^, SiO_4_^2−^, NO_3_^2-^, and PO_4_^3−^ anions at different ranges (0.1–0.50) mmol L^−1^ on the Cd(II) and Pd(II) adsorption by the ENMs is investigated and the results are presented in Fig. 8a, b. Each anion influenced the removal of Cd(II) and Pb(II) by the adsorbent. The effect of co-ions was examined on the Cd(II) and Pb(II) initial concentration at neutral pH. The experimental results indicate that Cl^−^ and F^−^ ions up to 0.1 mM and 0.5 mM concentration had minimal effect on Cd(II) adsorption, whereas SiO_4_^2−^, NO_3_^2-^, and PO_4_^3−^ exhibited some level of negative effect on Cd(II) adsorption. Cl^−^, SiO_4_^2−^, NO_3_^2-^, PO_4_^3−^ and F^−^ anions up to 0.1 mM and 0.5 mM concentration slightly affected the Pb(II) adsorption, which is consistent with published results^70,73^. The adsorption limit of SP-5 ENMs toward Cd(II) and Pb(II) blended with five anions is 90% and 70% lower than without anions or in the distilled water, as shown in Fig. 8a, b. These values have demonstrated excellent results compared with previous studies^73,74^. It affirms that the adsorption execution of SP-5 ENMs is effective and efficient and generally considered high even under extreme surrounding conditions.

### Regeneration and reuse

The recycle and reuse performance of the SP-5 ENMs were examined for 10 cycles to assess the suitability of reuse of the developed ENMs adsorbent and the obtained results are presented in Fig. 8c. It can be seen that the heavy metal binding capacity of the ENMs adsorbent gradually decreased with the higher number of recycling and reuse, which is obvious for any adsorbent. It is difficult to desorb 100% of the adsorbed heavy metals by the alkali regeneration method and therefore slowly the adsorbent losses its heavy metal ion binding capacity. The developed adsorbent showed slightly better binding of Cd(II) than Pb(II), although both have the same valency. After the first recycle and reuse, the removal of Cd(II) by the SP-5 ENMs was 93.3% and after 5 and 10 cycles, reduced to 73.7 and 56.5% respectively. For the removal of Pb(II), the corresponding values after 1, 5, and 10 cycles were 89, 77.2, and 49.4% respectively. The results show that the developed adsorbent can be recycled and reused at least five times.

### Density functional theory analysis

**Figure 9:**
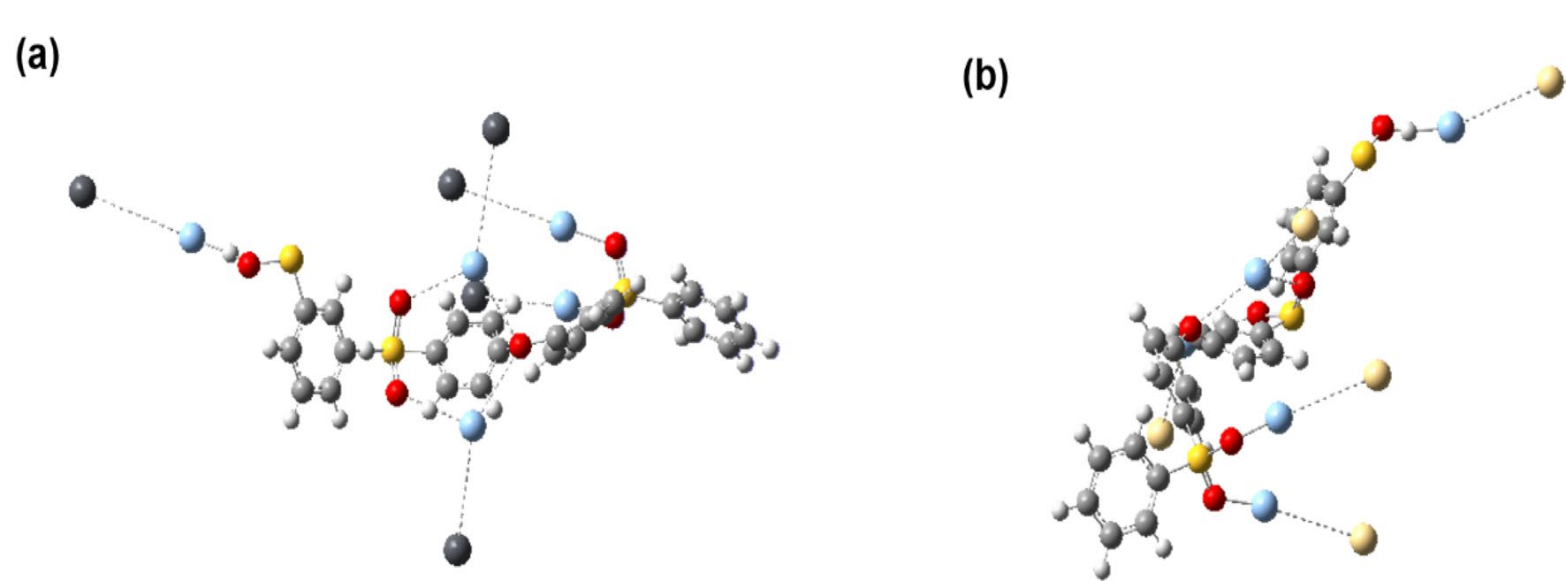
(**a**) Cd(II), and (**b**) Pb(II) binding to SP- 5 ENMs**.** Pb(II) and Cd(II) is depicted in deep grey color, sulfonate groups in magenta color, oxygen in red color, Ag in light green, and S in yellow color.

DFT calculations were performed using the Gaussian 6 software to verify the experimental selectivity and to further examine the chemical interactions between Cd(II), Pb(II), ions, and the EUOL@AgNPs-SP ENMs absorbent^75^. We used a piece of cross-linked SPES and PES as a model for computational information to mimic chemical selectivity. The anionic hydroxyl and sulfonate play a great role in binding heavy metal ions. The sulfonate groups are shown to bear more negative partial charges than hydroxyl groups in a Mulliken charge analysis (Fig. 9), and thus the sulfonate groups are expected to be a more suitable site for metal cation adsorption than hydroxyl groups. Figure 9 shows the optimized geometries of the cluster models SP M^2+^, in which the cation, M^2+^ (M = Pb II, Cd(II), is bonded to sulfonate in an optimized framework. The binding energy (E_bd_) values are measured as E_bd_ and shown in Fig. 9. A positive E_bd_ value means that the adsorption is favorable and that the relationship is stable^76^. The measured binding energies for Cd(II), Pb(II), are 219.35 and 206.26 kcal/mol respectively. As a result of the DFT measurements, it is revealed that SP-5 ENMs have the highest adsorption selectivity against Cd(II) of all of the heavy metal ions studied, as shown in Fig. 9a. These findings are consistent with Pearson's HSAB theorem, which predicts that borderline cations Zn(II) and Pb(II) will interact more strongly with the hard anion (O), whereas soft cations (Cd(II) and Hg(II) will interact the least.

In general, as the average M(II)O bond gap decreases, the interaction between metal cations and the oxygen atom of SP-5 ENMs becomes greater. Therefore, single-point energy (SPES) scans for all four metal cations were performed. The corresponding M(II)O bond distances are plotted in Fig. 9b, with the inset in Fig. 9b pictorially representing Pb(II) ion binding. The metal–oxygen distance increases in the order Pb(II), Cd(II), in SPE scan results; thus, Pb(II) ions interact the most at a comparatively short bond distance of 1.952 Å, while Cd(II) ions interact with the least at 2.35 Å. These findings also show that the Pb(II) ions and the SP-5 ENMs adsorbent have stronger and covalent interactions.

## Conclusion

We have successfully produced EUOL@Ag-NPs-incorporated SP nanocomposite ENMs by electrospinning with an average nanofiber diameter of 83.8 ± 21.54 nm and high hydrophilicity. TEM and SEM images reveal that EUOL@Ag-NPs were homogeneously dispersed in the SP nanofiber solution. With an increase in the EUOL@Ag-NPs loading, the modulus and tensile stress of ENMs increased to 10.05 MPa, a 95.2% improvement. The contact angle values of ENMs decreased from 15.7° for the SP-1 ENMs to 9.7° for SP-5 ENMs. SP-5 ENMs have shown excellent Cd(II) and Pb(II) binding capacity 625 mg g^−1^ and 370.37 mg g^−1^, respectively. Furthermore, the produced ENMs adsorbent have shown high heavy metal binding capacity even after 10 times recycle and reuse. The produced ENMs are a new effective adsorbent that may find end uses in the removal of heavy metals from potable water, and for cleaning our environment.

## Supplementary Information

Supplementary Information.

## Data Availability

The datasets generated during the current study are available from the corresponding author on reasonable request (Prof. Hongchen Song, H. Song).
