# Supplementary material for: Ag nanoparticles immobilized sulfonated polyethersulfone/polyethersulfone electrospun nanofiber membrane for the removal of heavy metals
Source: Sci Rep. 2022 Apr 6;12:5814. doi: 10.1038/s41598-022-09802-9 (PMC8986829; doi:10.1038/s41598-022-09802-9)
Supplement: Supplementary file 1 — Supplementary Information. [file 41598_2022_9802_MOESM1_ESM.docx]

**Supporting Information**

**Ag nanoparticles immobilized sulfonated polyethersulfone/polyethersulfone electrospun nanofiber membrane for the removal of heavy metals**

Md Eman Talukder^1,2,3,7#^, Md. Nahid Pervez^4#^, Wang Jianming^3^, George K Stylios^5^, Mohammad Mahbubul Hassan^6^, Hongchen Song^1,2,3*^, Vincenzo Naddeo^4*^, Alberto Figoli^7^

^1^ Shenzhen Institute of Advanced Technology, Chinese Academy of Sciences, Shenzhen, 518055, China

^2^ University of Chinese Academy of Sciences, Beijing, 100049, China

^3^ Guangdong Key Lab of Membrane Material and Membrane Separation, Guangzhou Institute of Advanced Technology, Guangzhou 511458, China

^4^ Sanitary Environmental Engineering Division (SEED), Department of Civil Engineering, University of Salerno, via Giovanni Paolo II 132, 84084 Fisciano (SA), Italy

^5^Research Institute for Flexible Materials, School of Textiles and Design, Heriot-Watt University, Galashiels, TD1 3HF, UK

^6^Fashion, Textiles and Technology Institute, University of the Arts London, 20 John Prince’s Street, London W1G 0BJ, UK

^7^Institute on Membrane Technology (CNR-ITM), University of Calabria, 87036 Rende (CS), Italy

*^#^ These authors contributed equally to the work*

**^*^ Corresponding authors:**

*Prof. Hongchen Song (hc.song@giat.ac.cn)*

*Prof. Vincenzo Naddeo (vnaddeo@unisa.it)*


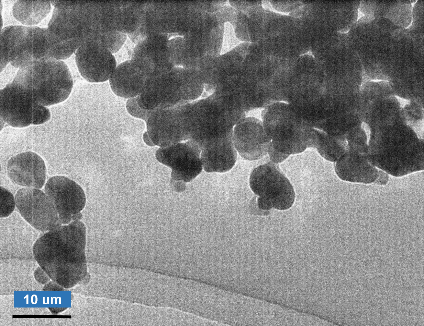

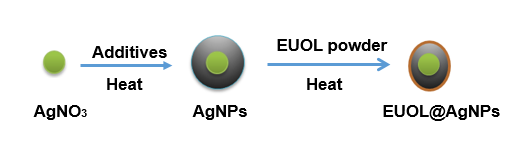


**Figure S1.** Synthesis of EUOL@Ag-NPs


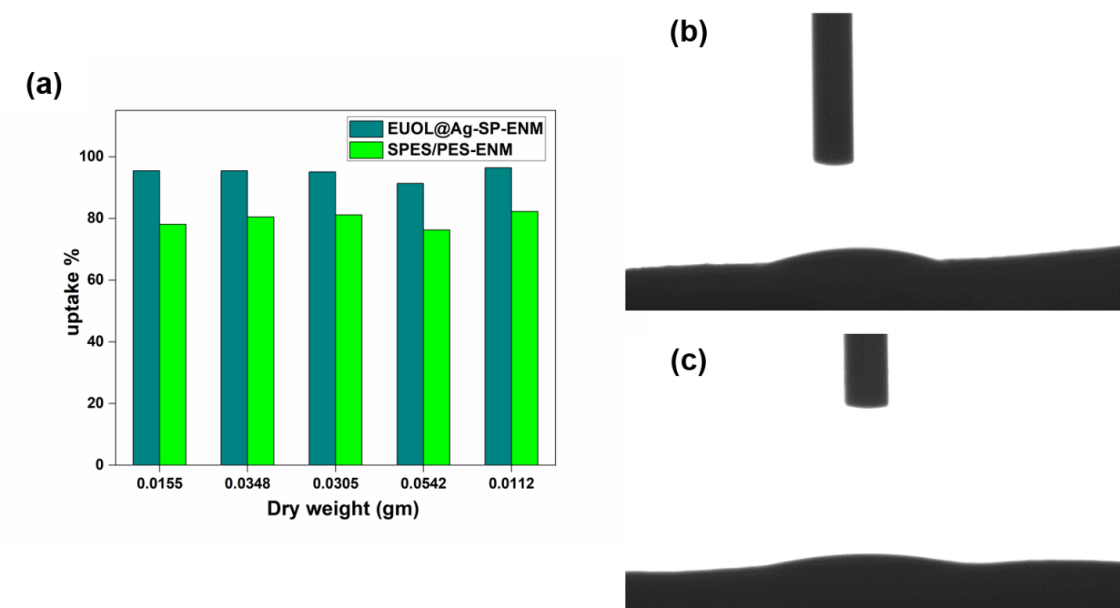


**Figure S2.** Super hydrophilic behavior (a) Water Uptake percentage of SP-1 and SP-5 ENMs, (b) water contact angle of SP-1, and (c) SP-5 ENMs





**Figure S3.** Calibration curve of heavy metal for different concentrations using ICP-OES

**EUOL@AgNPs stability studies**

As shown in Figure S4a and S4b; various NaCl concentrations (1 mM, 0.5 M, and 1 M) and different temperature (25–45°C and 70–90°C) were applied to study their effects, and EUOL@AgNP samples were examined by UV-Vis spectrophotometric analysis (UV-6-double beam spectrophotometer, Shanghai Lianhua Industrial Co., Ltd., China). On comparison of the UV-Vis spectra of the samples, the EUOL@AgNPs stability was decreased with an increase in the temperature and salt concentration^1^. Different NaCl stresses affected the synthesized EUOL@AgNPs, as shown in Figure S4. EUOL@AgNPs have shown less stability at 1 M NaCl, comparatively moderate stability at 0.5 M, and highest stability at 1 mM NaCl. The stability of EUOL@AgNPs at high temperatures was assessed by heating samples at different (25–45°C and 70–90°C) temperature ranges. EUOL@AgNPs were comparatively more stable at lower temperatures than higher temperatures. Also reported a decrease in UV-Vis light absorbance at higher temperatures is attributed to EUOL@AgNPs degradation in solution^2^.


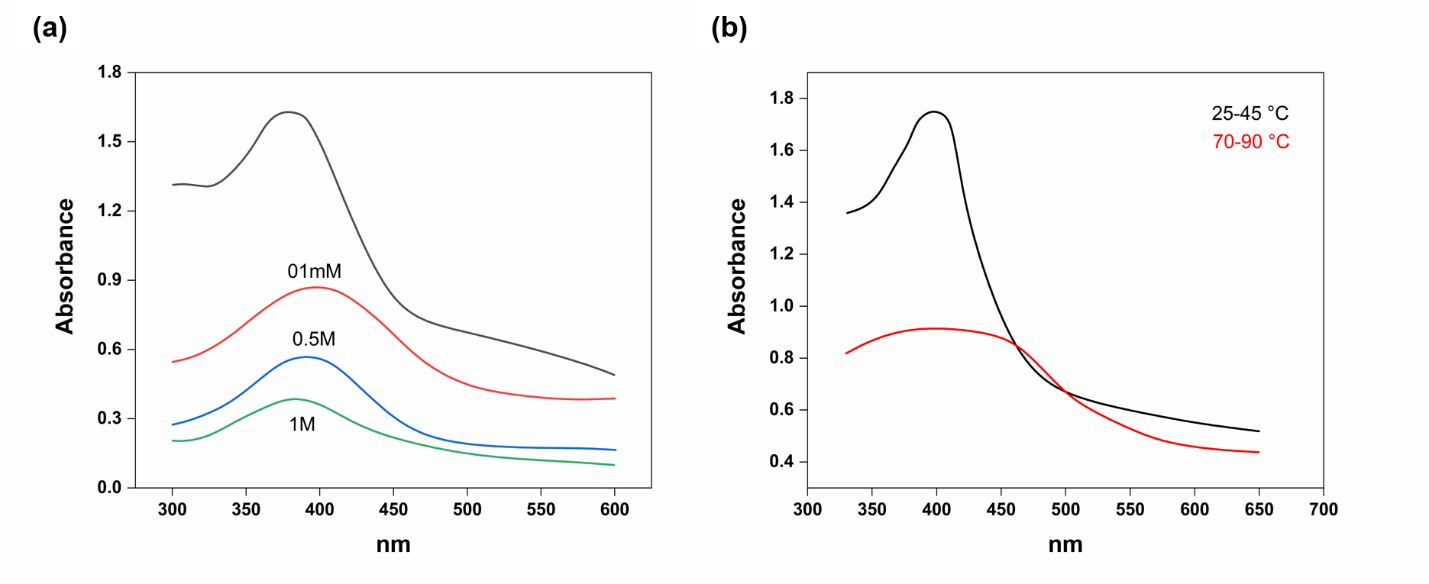


**Figure S4:** Comparison of UV-Vis spectra of EUOL@AgNPs stability at various NaCl concentrations (a) and different temperatures (b)

The zeta potential of EUOL@AgNPs synthesized from EUOL strain was found to be -4.1 mV which highlights the stability of the synthesized nanoparticles (Figure S5).





**Figure S5:** Zeta potential graph of EUOL@AgNPs (strain -4.1 mV) at neutral pH

Reference

1 Hasan, K. F., Horváth, P. G., Kóczán, Z. & Alpár, T. Thermo-mechanical properties of pretreated coir fiber and fibrous chips reinforced multilayered composites. *Sci. Reports* **11**, 1-13, doi:<https://doi.org/10.1038/s41598-021-83140-0> (2021).

2 Iqbal, M. *et al.* Green synthesis of silver nanoparticles from Valeriana jatamansi shoots extract and its antimicrobial activity. *Green Process. Synth.* **9**, 715-721, doi:<https://doi.org/10.1515/gps-2020-0066>.
